# Supplementary material for: Improving public support for climate action through multilateralism
Source: Nat Commun. 2022 Oct 28;13:6441. doi: 10.1038/s41467-022-33830-8 (PMC9616826; doi:10.1038/s41467-022-33830-8)
Supplement: Supplementary file 1 — Supplementary Information [file 41467_2022_33830_MOESM1_ESM.pdf]

Supplementary Information

Improving Public Support for Climate Action Through Multilateralism

Michael M. Bechtel, Kenneth F. Scheve, Elisabeth van Lieshout

Correspondence to: m.bechtel@uni-koeln.de, kenneth.scheve@yale.edu

Supplementary Figures 9
Supplementary Tables 1

Supplementary Tables

	Population	Raw Sample	Weighted Sample
France			
Age: 18-34	20	26	20
Age: 35-49	23	24	23
Age: 50-64	32	28	32
Age: 65+	26	22	25
Education: 16 yrs or less	26	12	26
Education: 17-18	25	48	25
Education: 19+	49	40	49
Gender: Male	47	45.8	47
Gender: Female	53	54.2	53
Germany			
Age: 18-34	19	25	19
Age: 35-49	21	22	21
Age: 50-64	35	29	35
Age: 65+	24	24	25
Education: 16 yrs or less	38	43	38
Education: 17-18	19	32	19
Education: 19+	42	25	43
Gender: Male	49	49	49
Gender: Female	51	51	51
United Kingdom			
Age: 18-34	22	27	22
Age: 35-49	26	28	26
Age: 50-64	30	22	30
Age: 65+	22	23	22
Education: 16 yrs or less	41	33	41
Education: 17-18	28	21	28
Education: 19+	31	47	21
Gender: Male	50	46	50
Gender: Female	50	54	50
United States			
Age: 18-34	30	27	30
Age: 35-49	25	22	25
Age: 50-64	25	29	25
Age: 65+	20	22	20
Education: Less than High School	12	7	12
Education: High School Degree	28	29	28
Education: Some College	31	32	31
Education: BA or higher	29	32	29
Gender: Male	48	47	49
Gender: Female	51	53	51

Supplementary Table 1. Distribution of sociodemographics in the population, the raw sample, and the weighted sample (by country) (Total N=10,081). Source for the population margins are the 2016 American Community Survey for the United States and the 2018 Eurobarometer for France, Germany, and the United Kingdom.

Supplementary Figures

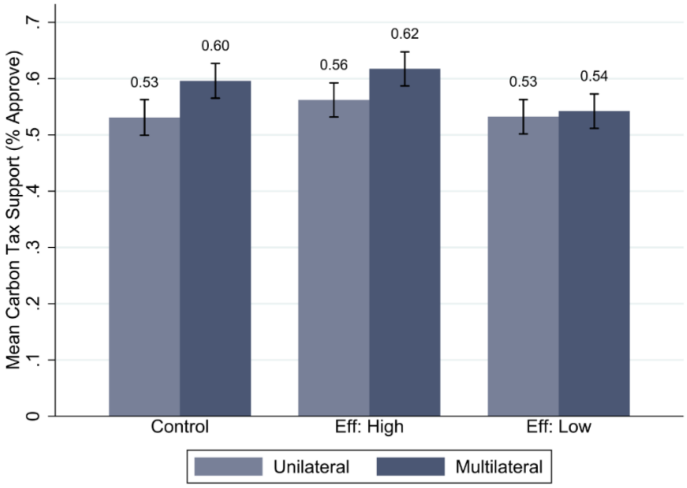

a Proportion Supporting a Carbon Tax by Multilateralism and Effectiveness Prime

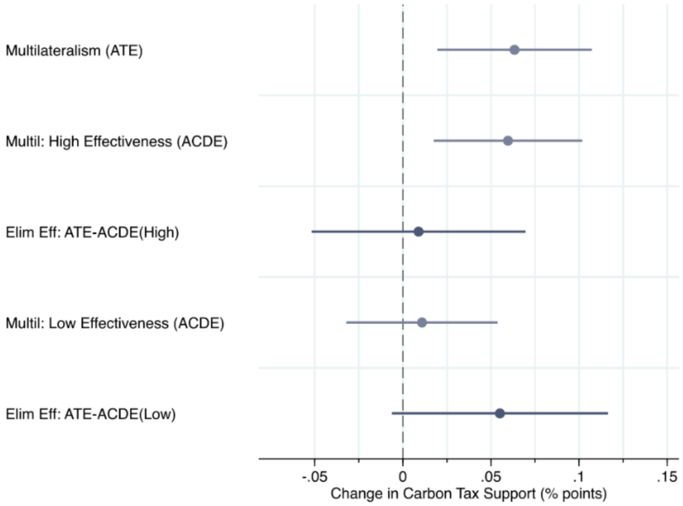

b Causal and Eliminated Effects (EE) of Multilateralism by Effectiveness Prime

Supplementary Fig. 1 | The Effect of Multilateralism on Carbon Tax Support (N=6,000), Unweighted. (a) The figure shows the proportion of individuals supporting the introduction of a carbon tax by randomly assigned multilateralism and effectiveness conditions. **(b)** The figure reports the causal effects of multilateralism by randomly assigned effectiveness condition (control=no information, Eff: High=High Effectiveness, Eff: Low=Low Effectiveness) along with the eliminated effects (EE) estimated using a linear probability model with robust standard errors. Regressions control for gender, age, income, education,and employment status. Country fixed effects included. Error bars indicate 95% confidence intervals. N(France)=2,000, N(Germany)=2,000, N(United Kingdom)=2,000.

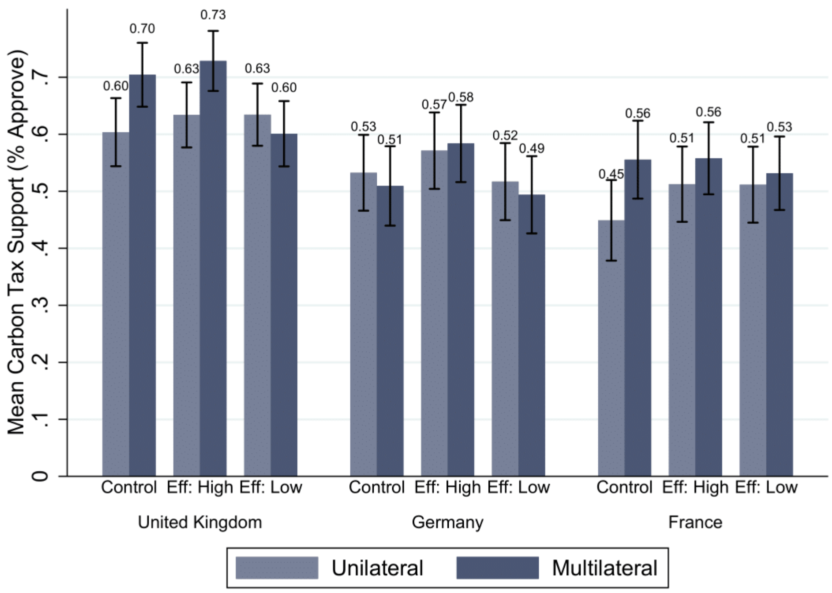

a Proportion Supporting a Carbon Tax by Multilateralism and Effectivenss Prime

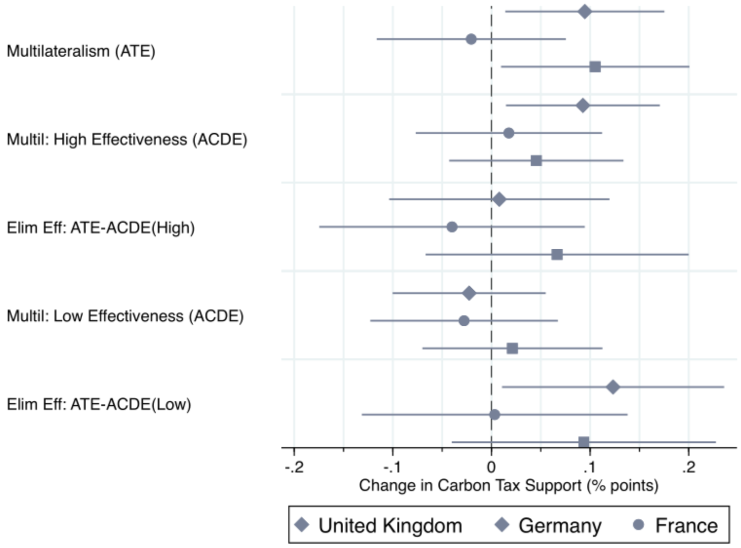

b Causal Effects of Multilateralism by Effectiveness Prime

Supplementary Fig. 2| The Effect of Multilateralism on Carbon Tax Support (N=6,000), by Country. **(a)**The figure shows the proportion of individuals supporting the introduction of a carbon tax by randomly assigned multilateralism and effectiveness conditions. **(b)** The figure reports the causal effects of multilateralism by randomly assigned effectiveness condition (control=no information, Eff: High=High Effectiveness, Eff: Low=Low Effectiveness) estimated using a linear probability model with robust standard errors. Error bars indicate 95% confidence intervals. Survey weights applied. N(France)=2,000, N(Germany)=2,000, N(United Kingdom)=2,000.

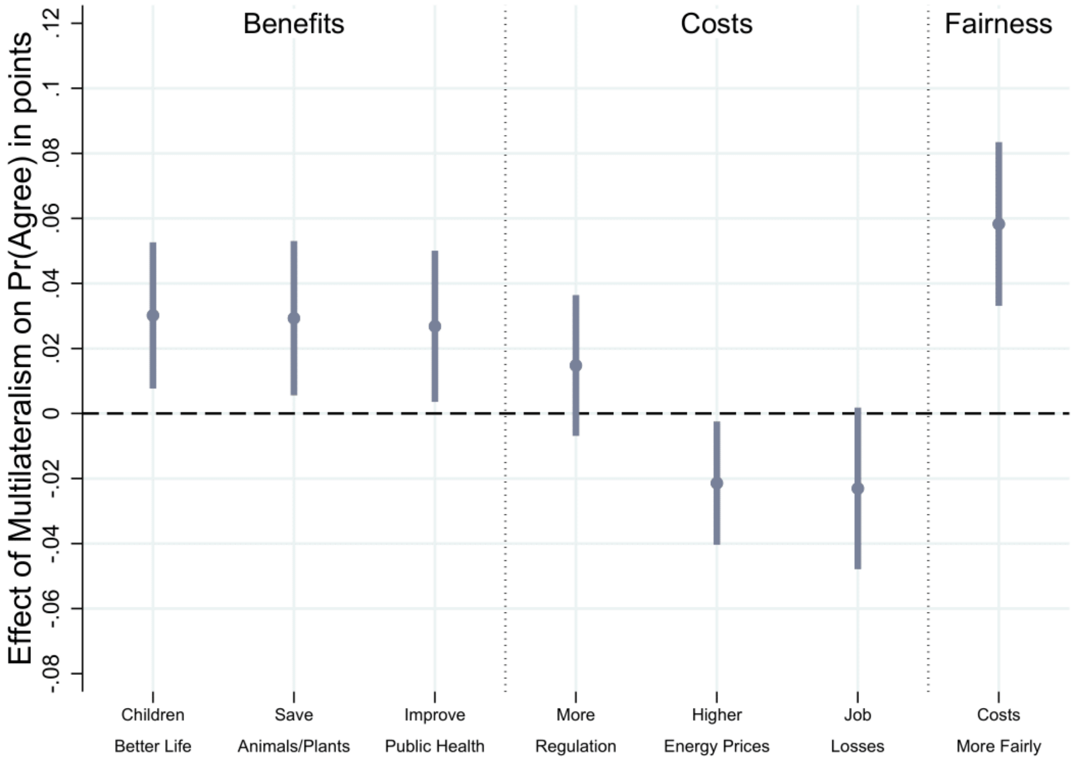

Supplementary Fig. 3 | The Causal Effects of Multilateralism on Expectations about the Benefits, Costs, and Fairness of Climate Action in France, Germany, and the United Kingdom (N=6,000), Unweighted. This plot reports coefficients from linear regressions of statement approval on a binary indicator that is one if climate action is multilateral and is zero if climate action is unilateral. Error bars indicate 95% confidence intervals. Without use of survey weights. N(France)=2,000, N(Germany)=2,000, N(United Kingdom)=2,000.

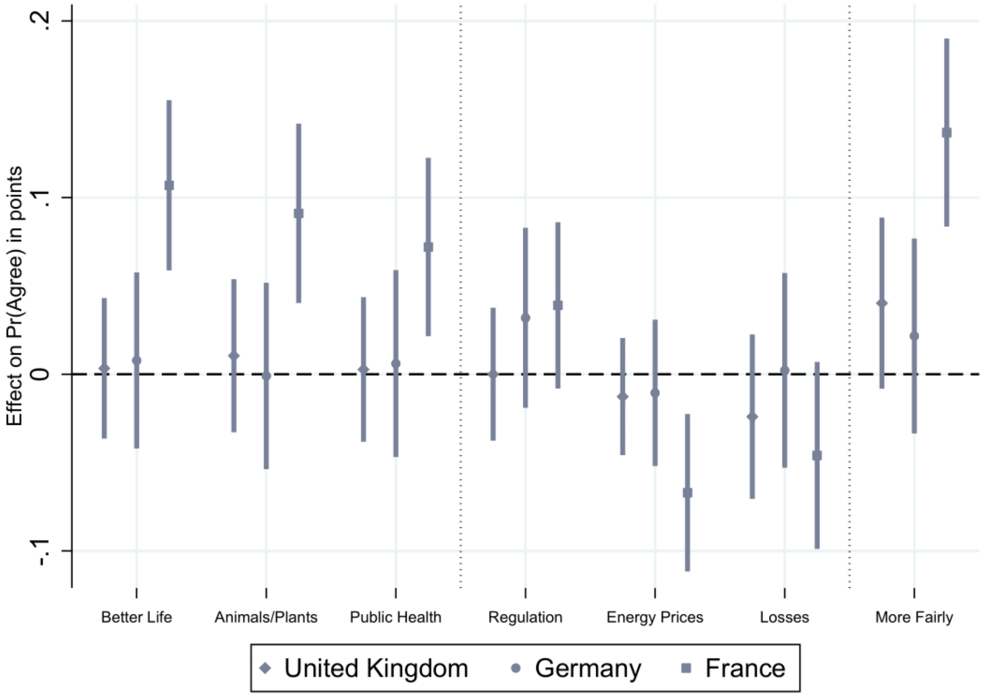

Supplementary Fig. 4 | The Causal Effects of Multilateralism on Expectations about the Benefits, Costs, and Fairness of Climate Action in France, Germany, and the United Kingdom (N=6,000), by Country. This plot reports coefficients from linear regressions of statement approval on a binary indicator that is one if climate action is multilateral and is zero if climate action is unilateral. Error bars indicate 95% confidence intervals. Survey weights applied. N(France)=2,000, N(Germany)=2,000, N(United Kingdom)=2,000.

We will now provide you with several scenarios which describe a set of policies for the United States that will impact climate change and information about what other major economies are doing. The scenarios will vary in how costly they are to households, how those costs will change over time, whether investments will be made in mitigation efforts to reduce greenhouse gas emissions thus making global warming less likely or in adaptation efforts to adjust to environmental change to lessen the negative effects of global warming. These dimensions will vary both for the United States and in other major economies.

For each comparison we would like you to tell us which of the scenarios you prefer. You may like several alternatives similarly or may not like either of them at all. Regardless of your overall evaluation, please indicate which alternative you prefer.

In total, we will show you 8 comparisons. People have different opinions about this issue and there are no right or wrong answers. Please take your time when reading the potential scenarios.

	**Scenario 1**	**Scenario 2**
**In the United States**		
Average household costs per month	$107	$213
Distribution of costs over time	gradually decreasing	constant over time
Adaptation (new technology, building dams, …)	88%	2%
Mitigation (reducing greenhouse gas emissions, removing greenhouse gases from the atmosphere)	12%	98%
**In other major economies**		
Average household costs per month	$53	$107
Distribution of costs over time	gradually decreasing	gradually decreasing
Adaptation (new technology, building dams, …)	0%	62%
Mitigation (reducing greenhouse gas emissions, removing greenhouse gases from the atmosphere)	100%	38%
Which of these scenarios do you prefer?	o	o

Supplementary Fig. 5 | Instructions and Example Profile for the Climate Multilateralism Conjoint Experiment. This figure shows the instructions to respondents for the conjoint experiment and an example profile pair presented to respondents in the United States. Respondents assessed eight paired profiles. The experiment randomly varied whether the attributes for a respondent's own country or those for other major economies were listed first. For each respondent the order remained unchanged for all conjoint tasks. All attribute levels were fully and separately randomized.

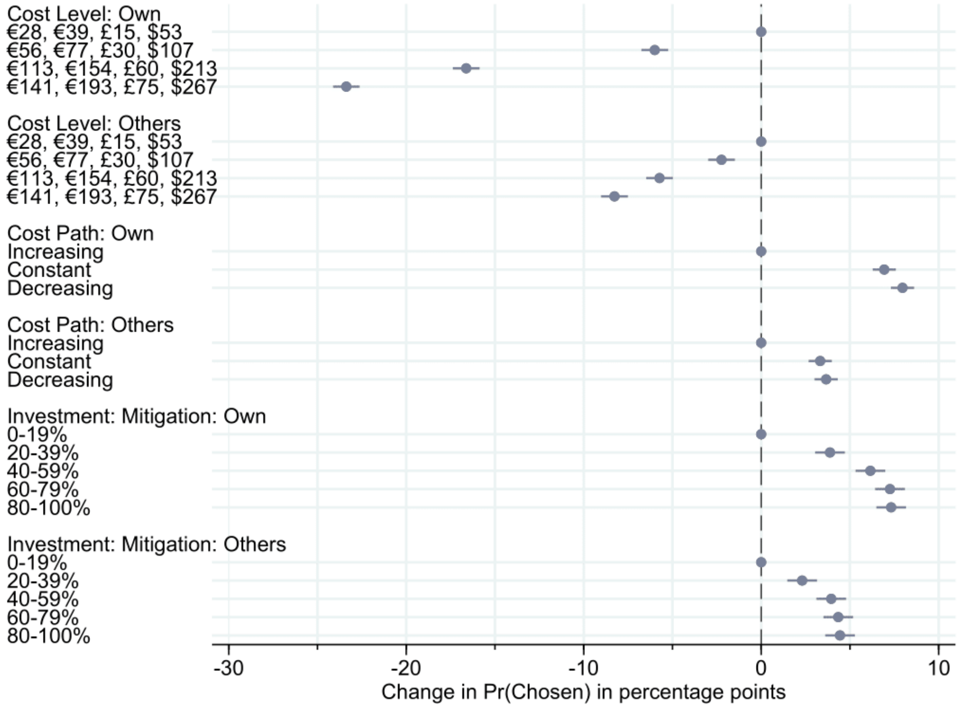

Supplementary Fig. 6 | The Causal Effects of Climate Policy Choices by Other Countries on Public Support in France, Germany, United Kingdom, and the United States (N=129,280), Unweighted. This plot reports coefficients from linear regressions of policy approval on randomly assigned climate policy choices made by other countries. Error bars indicate 95% confidence intervals. Without use of survey weights. N(France)=32,000, N(Germany)=32,000, N(United Kingdom)=32,000, N(United States)=33,280.

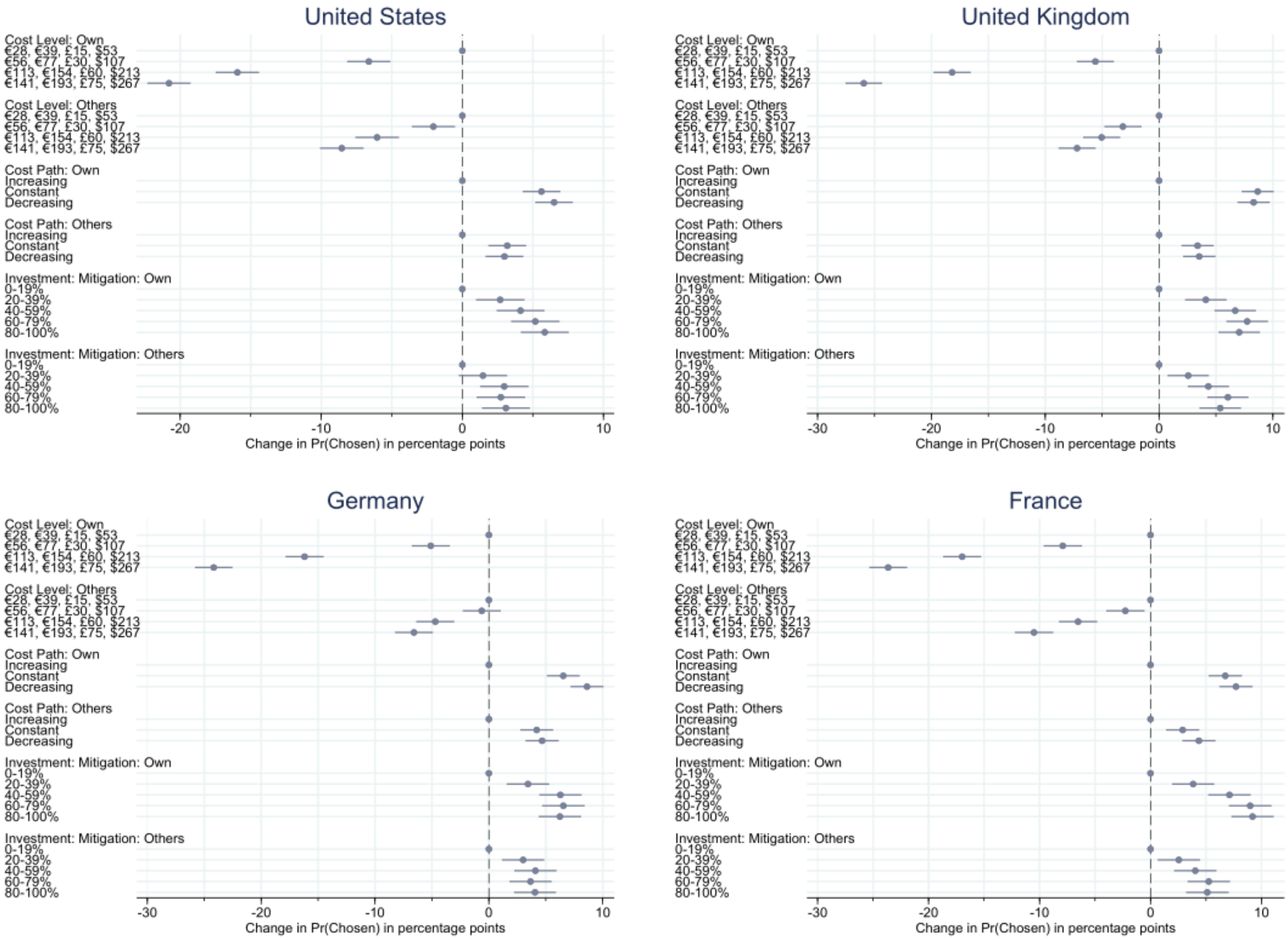

Supplementary Fig. 7 | The Causal Effects of Climate Policy Choices by Other Countries on Public Support in France, Germany, United Kingdom, and the United States (N=129,280), by country. This plot reports coefficients from linear regressions of policy approval on randomly assigned climate policy choices made by other countries. Error bars indicate 95% confidence intervals. Survey weights applied. N(France)=32,000, N(Germany)=32,000, N(United Kingdom)=32,000, N(United States)=33,280.

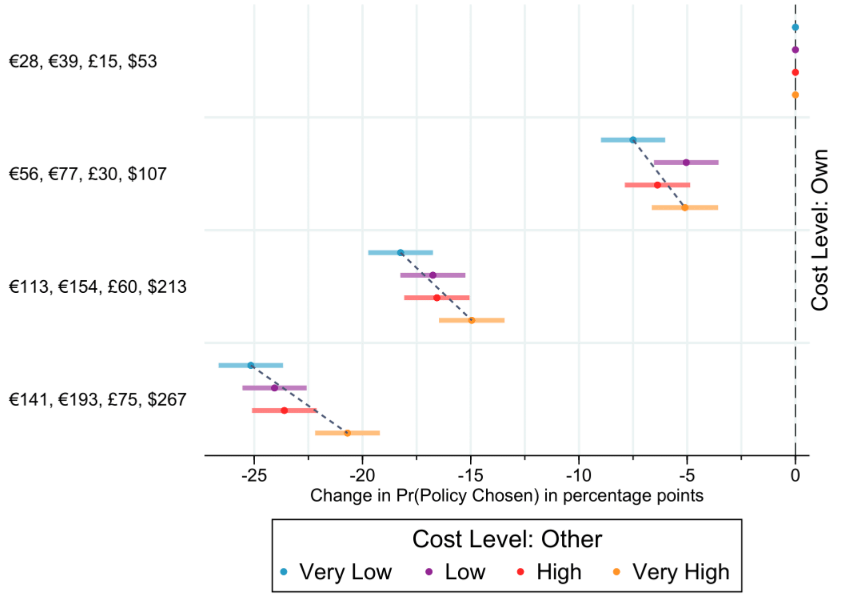

a Effects of Own Cost, Conditional on Other Costs

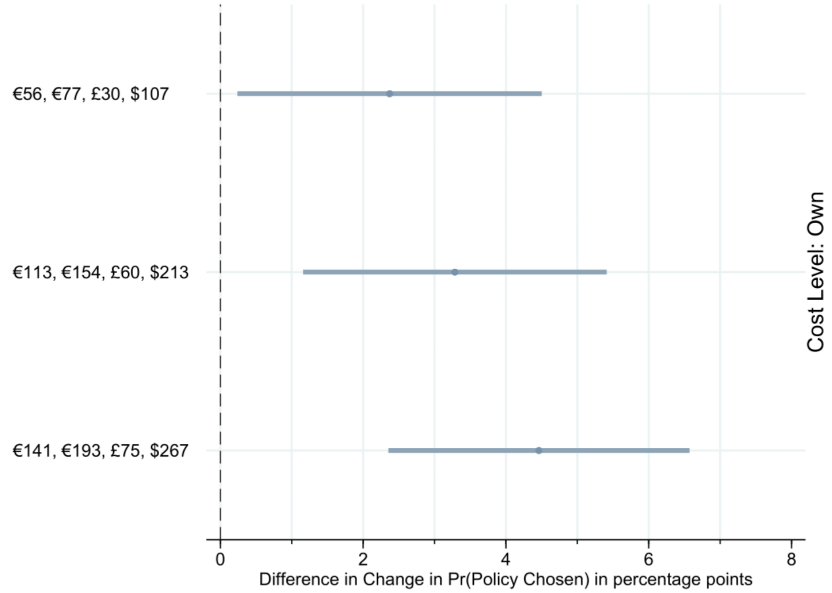

b Difference in Effect of Own Cost if Other Costs Increase from Very Low to Very High

Supplementary Fig. 8 | The Causal Effects of Climate Policy Costs by Other Costs in France, Germany, United Kingdom, and the United States (N=129,280), Unweighted. This plot reports coefficients from linear regressions of policy approval on randomly assigned climate policy household costs introduced in other countries. Error bars indicate 95% confidence intervals. Without use of survey weights. N(France)=32,000, N(Germany)=32,000, N(United Kingdom)=32,000, N(United States)=33,280.

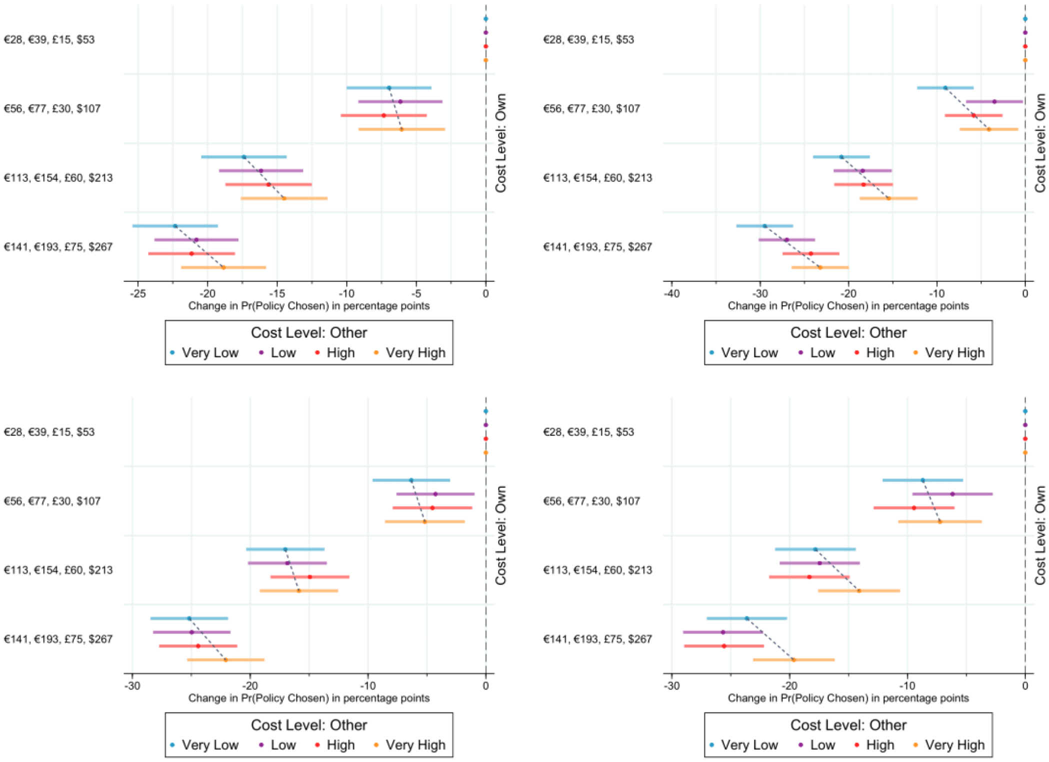

a Effects of Own Cost, Conditional on Other Costs

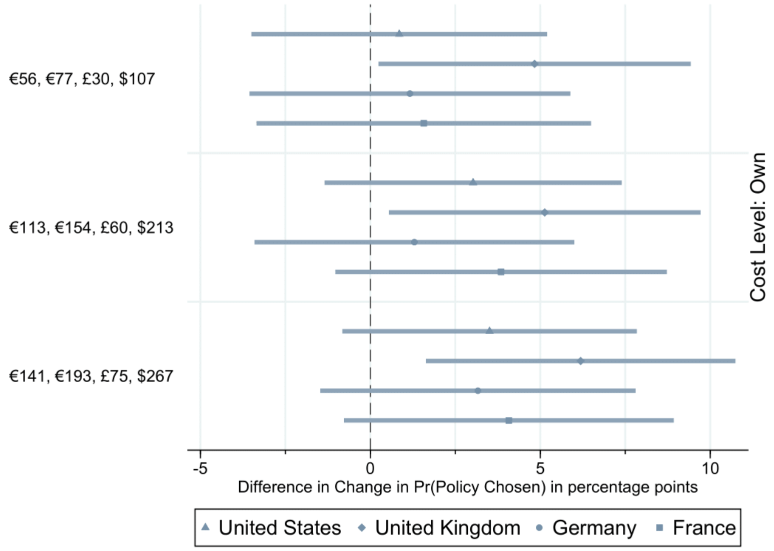

b Difference in Effect of Own Cost if Other Costs Increase from Very Low to Very High

Supplementary Fig. 9 | The Causal Effects of Climate Policy Costs by Other Costs in France, Germany, United Kingdom, and the United States (N=129,280), by country. This plot reports coefficients from linear regressions of policy approval on randomly assigned climate policy household costs introduced in other countries. Error bars indicate 95% confidence intervals. Without use of survey weights. N(France)=32,000, N(Germany)=32,000, N(United Kingdom)=32,000, N(United States)=33,280.
