# Supplementary material for: Improving public support for climate action through multilateralism
Source: Nat Commun. 2022 Oct 28;13:6441. doi: 10.1038/s41467-022-33830-8 (PMC9616826; doi:10.1038/s41467-022-33830-8)
Supplement: Supplementary file 3 — Reporting Summary [file 41467_2022_33830_MOESM3_ESM.pdf]

nature portfolio

Corresponding author(s): Michael M. Bechtel
Kenneth F. Scheve

Last updated by author(s): Sep 13, 2022

# Reporting Summary

Nature Portfolio wishes to improve the reproducibility of the work that we publish. This form provides structure for consistency and transparency in reporting. For further information on Nature Portfolio policies, see our Editorial Policies and the Editorial Policy Checklist.

## Statistics

For all statistical analyses, confirm that the following items are present in the figure legend, table legend, main text, or Methods section.

| n/a | Confirmed | |
|---|---|---|
| ☐ | ☒ | The exact sample size (*n*) for each experimental group/condition, given as a discrete number and unit of measurement |
| ☐ | ☒ | A statement on whether measurements were taken from distinct samples or whether the same sample was measured repeatedly |
| ☐ | ☒ | The statistical test(s) used AND whether they are one- or two-sided<br>*Only common tests should be described solely by name; describe more complex techniques in the Methods section.* |
| ☐ | ☒ | A description of all covariates tested |
| ☐ | ☒ | A description of any assumptions or corrections, such as tests of normality and adjustment for multiple comparisons |
| ☐ | ☒ | A full description of the statistical parameters including central tendency (e.g. means) or other basic estimates (e.g. regression coefficient) AND variation (e.g. standard deviation) or associated estimates of uncertainty (e.g. confidence intervals) |
| ☐ | ☒ | For null hypothesis testing, the test statistic (e.g. *F*, *t*, *r*) with confidence intervals, effect sizes, degrees of freedom and *P* value noted<br>*Give P values as exact values whenever suitable.* |
| ☒ | ☐ | For Bayesian analysis, information on the choice of priors and Markov chain Monte Carlo settings |
| ☒ | ☐ | For hierarchical and complex designs, identification of the appropriate level for tests and full reporting of outcomes |
| ☐ | ☒ | Estimates of effect sizes (e.g. Cohen's *d*, Pearson's *r*), indicating how they were calculated |

*Our web collection on statistics for biologists contains articles on many of the points above.*

## Software and code

Policy information about availability of computer code

| | |
|---|---|
| Data collection | Excel (MacOS 16.65), Stata 16, R |
| Data analysis | Excel (MacOS 16.65), Stata 16, R |

For manuscripts utilizing custom algorithms or software that are central to the research but not yet described in published literature, software must be made available to editors and reviewers. We strongly encourage code deposition in a community repository (e.g. GitHub). See the Nature Portfolio guidelines for submitting code & software for further information.

## Data

Policy information about availability of data

All manuscripts must include a data availability statement. This statement should provide the following information, where applicable:

- Accession codes, unique identifiers, or web links for publicly available datasets
- A description of any restrictions on data availability
- For clinical datasets or third party data, please ensure that the statement adheres to our policy

The data used in this study are available at the Harvard Dataverse as Bechtel, Michael; Scheve, Kenneth; van Lieshout, Elisabeth, 2022, "Replication Data for: Improving Public Support for Climate Action Through Multilateralism", https://doi.org/10.7910/DVN/GCNO77, Harvard Dataverse, V1. The following software has been used to process the data: Excel (MacOS 16.65), Stata 16, and R.

# Field-specific reporting

Please select the one below that is the best fit for your research. If you are not sure, read the appropriate sections before making your selection.

☐ Life sciences ☒ Behavioural & social sciences ☐ Ecological, evolutionary & environmental sciences

For a reference copy of the document with all sections, see nature.com/documents/nr-reporting-summary-flat.pdf

# Behavioural & social sciences study design

All studies must disclose on these points even when the disclosure is negative.

| | |
|---|---|
| Study description | Quantitative and qualitative survey data. |
| Research sample | Samples of adult population in four major industrialized countries (France, Germany, UK, US). |
| Sampling strategy | Quota sampling in combination with matched sampling using propensity scores. The samples are designed to be representative of the adult population in each country. The propensity score function included age, gender, race/ethnicity, years of education, and region. Sociodemographic distributions by country are reported in Supplementary Table 1. The propensity scores were grouped into deciles of the estimated propensity score in the frame and post-stratified according to these deciles. The sample size follows the existing literature on this topic. The country selection is driven by the goal of studying advanced democracies that are also Annex 1 countries. |
| Data collection | The data was collected through anonymous online surveys with blinded treatment conditions. No researcher was present during the surveys. |
| Timing | Dec 2018 to April 2019. |
| Data exclusions | No data excluded. |
| Non-participation | No participants dropped out. |
| Randomization | Random assignment into experimental conditions. |

# Reporting for specific materials, systems and methods

We require information from authors about some types of materials, experimental systems and methods used in many studies. Here, indicate whether each material, system or method listed is relevant to your study. If you are not sure if a list item applies to your research, read the appropriate section before selecting a response.

## Materials & experimental systems

| n/a | Involved in the study | |
|---|---|---|
| ☒ | ☐ | Antibodies |
| ☒ | ☐ | Eukaryotic cell lines |
| ☒ | ☐ | Palaeontology and archaeology |
| ☒ | ☐ | Animals and other organisms |
| ☐ | ☒ | Human research participants |
| ☒ | ☐ | Clinical data |
| ☒ | ☐ | Dual use research of concern |

## Methods

| n/a | Involved in the study | |
|---|---|---|
| ☒ | ☐ | ChIP-seq |
| ☒ | ☐ | Flow cytometry |
| ☒ | ☐ | MRI-based neuroimaging |

## Human research participants

Policy information about studies involving human research participants

| | |
|---|---|
| Population characteristics | See above. |
| Recruitment | Respondents were recruited through YouGov drawing on their opt-in panels. The sampling and matching procedure (see above) was applied to counteract potential self-selection. |
| Ethics oversight | Informed consent was obtained from all participants. The study was approved by the IRBs at Washington University in St. Louis (#201803178) and Stanford University (eProtocol 46325). |

Note that full information on the approval of the study protocol must also be provided in the manuscript.
